# Clinical Comparison of the Volumetric Changes in Single Pontic Site Development through Connective Tissue Grafting Using Modified Pouch Technique versus Pouch Technique in the Maxillary Esthetic Zone: A Randomized Controlled Clinical Trial

**DOI:** 10.1155/2022/1677471

**Published:** 2022-08-25

**Authors:** Ahmed H. Ammar, Enji Ahmed, Ahmed ElBarbary, Dalia Ghalwash, Azza Ezz Elarab

**Affiliations:** ^1^Oral Medicine and Periodontology Department, Faculty of Dentistry, Postal Code: 11553, Cairo University, Cairo, Egypt; ^2^Oral Medicine and Periodontology Department, Faculty of Dentistry, The British University in Egypt, Postal Code: 11837, El- Sherouk, Egypt; ^3^Oral Medicine and Periodontology Department, Faculty of Dentistry, Al-Ahram Canadian, 4th Industrial Zone, Banks Complex, 6th of October City (2), Giza Governorate 12563, Egypt

## Abstract

**Aim:**

The aim is to compare the volumetric changes between pouch technique versus the modified pouch technique in pontic site development using connective tissue graft in patients that have Seibert class I ridge defects in the maxillary esthetic zone. *Methodology*. This randomized, controlled, double-blinded, parallel-grouped clinical trial included sixteen patients with a single pontic site in the maxillary esthetic area presenting Seibert Class I ridge defects. Patients were randomly assigned into two equal groups: test group (n: 8) received soft tissue augmentation with connective tissue graft using the modified pouch technique and control group (n: 8) received soft tissue augmentation with connective tissue graft using pouch technique. The volumetric evaluation was carried out by taking impressions at baseline, 3 and 6 months after the surgery. Keratinized tissue thickness was also measured at baseline, 3 and 6 months after the surgery. Visual analogue scale (VAS) was recorded by the patients at day 3, day 7, and day 14 after the surgery.

**Results:**

The test group had more increase in soft tissue volume than the control group at baseline, 3 months, and 6 months. The keratinized tissue width at baseline in the test group had a higher value than that of the control group. At 3 months, both groups had the same mean value, while at 6 months, the test group had a higher value than the control group. Regarding postoperative pain, the visual analogue scale shown at day 3 in the test group had a higher value than that of the control group, while at day 7, the control group had a higher value than the test group. At day 14, both groups had the same mean value.

**Conclusions:**

Soft tissue augmentation using both the traditional pouch technique and the modified pouch technique led to successful soft tissue volume augmentation in pontic site development in Seibert Class I ridge defects with no statistically significant difference between the two techniques.

## 1. Introduction

Immediately following tooth extraction, biological processes are initiated, which can lead to substantial resorptive processes of the alveolar ridge and lead to localized alveolar ridge defects, which result in the need for surgical reconstruction [1]. The replacement of missing teeth is mandatory for proper chewing of food, esthetic jaw support, and stability of the remaining teeth. Missing teeth disrupt proper function and the teeth next and opposite to the missing tooth/tooth will shift, move, and tip in space of time. It is better to restore a soon missing tooth than ending by the consequences of long-term postextraction complications [2].

The size and shape of the pontic have been also documented to contribute to some problems that occur with the natural tooth abutments. These problems are mainly related to the hindrance of sanitization by classic or special oral hygiene methods, which may cause accumulation of plaques under the bridge in the immediate vicinity of the abutments [3].

Surgical periodontal procedures are considered an integral component of the recent approach for the treatment of periodontal diseases. Soft tissue grafting has been increasingly used in clinical practice for augmenting tissue thickness, reestablishing an adequate width of keratinized tissue, correcting mucogingival deformities, and improving aesthetics, at teeth and dental implant sites [4, 5].

The augmentation of alveolar ridge defects can be indicated in various clinical situations to improve aesthetics, function, hygiene, and long-term tissue stability. In general, a lack of tissue can be reconstructed either at the bone level using bone augmentation procedures or at the soft tissue level using connective tissue grafts [6] Usually, bone augmentation procedures are performed in connection with implant site development or for the reconstruction of extensive ridge defects. However, for the development of pontic areas, sufficient soft tissue thickness is necessary, allowing for conditioning the pontic area [7]. Creating an appropriate soft tissue condition and pontic design improves function, aesthetics, and cleans ability [8].

The use of soft tissue grafts in the augmentation of ridge defects is a well-documented approach. Additionally, soft tissue volume augmentation procedures have been also proposed to surgically correct localized alveolar defects such as preprosthetic site development and soft tissue contouring around implants [9, 10]. Among soft tissue grafts, subepithelial connective tissue grafts (SCTG) are superior in terms of volume gain, aesthetics, and long-term stability compared with full-thickness free gingival grafts and are currently the gold standard of care for soft tissue volume augmentation procedure [4, 9, 11]. The results of preclinical and clinical studies have shown different techniques to be successful [7, 12–15]. Despite their successful clinical application, there is little knowledge regarding the long-term behavior of the grafts in the augmented area in terms of volume stability.

The tunnel technique was originally described for the treatment of gingival recession-type defects, and it is still predominantly perceived in this particular indication. Application of the technique demands advanced surgical training, and the use of specifically designed microsurgical instruments is strongly recommended [4]. Tunnelling flap procedures have developed into a truly multifunctional approach for soft tissue augmentation in the esthetic zone. Considering the benefits of incision-free flap elevation, the technique has been introduced to a considerably expanded range of indications. A modification of tunnelling flap procedures—the modified pouch technique—has proven to be a well-designed treatment modality for pontic site development [16]. The technique allows for surgical reconstruction of the alveolar ridge with definitive tissue sculpting around the provisional pontic in a single intervention. In doing so, the modified pouch technique combines the advantage of tunnelling flap preparation with the benefit of a substantially simplified and shortened surgical procedure when compared with conventional ways of alveolar ridge augmentation, such as the inlay grafting technique, for instance [16].

Accordingly, the present investigation was carried out to compare the pouch technique and the modified pouch technique in pontic site development using connective tissue graft in patients that have Seibert class I ridge defects in the maxillary esthetic zone in terms of volumetric changes, keratinized tissue thickness, and postoperative pain.

## 2. Subjects and Methods

The study protocol was approved by the Ethics Committee of Scientific Research, Faculty of Dentistry, Cairo University, in 19 February 2019, with approval number 19-2-26. The detailed operation and follow-up periods were clearly described in detail to all patients, and then all subjects participated in this trial, signed a written consent, and agreed to participate in this clinical trial. Subjects were selected from the outpatient clinic, Department of Oral Medicine and Periodontology, Faculty of Dentistry, Cairo University, between March 2019 and August 2021. Screening of patients was continued until the target sample was achieved. Identifying and recruiting potential subjects were achieved through the patients' database. All surgical procedures were carried out by only one operator (Ahmed H Ammar (first author)). This clinical trial was registered in U.S. National Institutes of Health Clinical Trials Registry, Clinicaltrials.gov ID: NCT03882216. Based on a previous study by Akali et al. [10], the calculated sample size was 16 (8 in each group), increased number of anticipated missing data: 22 (11 in each of the groups). The sample size calculation was achieved using PS program (power and sample size program: https://biostat.mc.vanderbilt.edu/twiki/bin/view/M/PowerSampleSize) and approved by medical biostatistics unit, Cairo University, in February 2019. The outcome that is used to calculate the sample size was volumetric changes (primary outcome) in 6 months interval. Parameters used for outcome were Mean difference and SD,which were not available in the study conducted by Akali et al. [10] and were estimated by 0.5 (It is the minimal clinically important difference as estimated by the expert (Prof. Azza Ezz El Arab (fifth author)). The Alpha level of significance was 0.05, and power of the study was 0.8, while the sample size was statistically calculated using T-test independent as 16 pontic sites (8 in each group) and increased number for anticipated missing data to 22 pontic sites (11 in each group).

Inclusion criteria included systemically free patients, patients with good oral hygiene with missing single tooth in the esthetic region, Seibert Class I ridge defect, and buccolingual loss of the ridge contour, and the healing period following tooth extraction had to be at least 3 months prior to the surgical procedure and palate with sufficient connective tissue graft to accommodate ridge augmentation.

Exclusion criteria included smokers, occlusal trauma at the site of the graft, pregnancy and lactation, severe gagging reflex, systemic conditions or medications that could alter soft tissue healing, and unwillingness to commit to follow needed visits or to provide written informed consent.

Pontic sites were randomly assigned with simple randomization, using computer-generated random numbers (Research Randomizer (Version 4.0) [Computer software], Retrieved on November 5, 2018, from http://www.randomizer.org/) done by the co-supervisor E.A. into two equal groups: Group I: pouch technique; and Group II: modified pouch technique; each group contained 11 pontic sites with a total of 22 pontic sites and a total of 16 patients. The connective tissue graft was harvested, and then the decision of which technique to be used in the pontic site was selected according to the randomized numbers in a sequentially numbered, opaque sealed envelope, and the number was picked by the main supervisor. This study was a double-blinded randomized clinical trial. Blinding of participants, outcome assessor, and biostatistician was achieved, while blinding of the operator was not possible.

### 2.1. Treatment Protocol

#### 2.1.1. Preoperative Assessment and Preparation

Full medical history and proper diagnosis of the ridge defects were done to decide the suitable treatment plan and to ensure that the patient was adapted to the inclusion and exclusion criteria. After the patient was accepted to be enrolled in this study, full mouth supra- and subgingival debridement was performed using ultrasonic device (Woodpecker UDS-P with LED, China) with supragingival scaling inserts (EMS Woodpecker ultrasonic scaler tip, Woodpecker, China) followed by Universal or Gracey's curettes (Nordent curettes; Nordent Manufacturing Inc, USA) for proper subgingival debridement. Patient preparation was completed in a single visit. Proper oral hygiene instructions were given to the patient including tooth brushing 2 times daily by soft toothbrush using a circular scrub technique and interdental cleansing using waxed dental floss or toothpick according to the size of the interdental embrasure. Chemical plaque control with 0.125% chlorhexidine HCL mouthwash (Hexitol00AE) (Hexitol®: Chlorhexidine HCL mouthwash, The Arab Drug Company for pharmaceutical and CHEM. IND. CO. Cairo-Egypt) was prescribed to be used twice daily for 2 weeks. Impressions were taken using high viscosity silicon impression material to introduce better details and high dimensional stability models (GC Exaflex) (GC Corporation, Tokyo, Japan). Models were cast in dental stone and used as the baseline reference for volumetric measurements (GC Fujirock type 4). One month and 3 and 6 months after the surgical volume augmentation procedure, clinical periodontal measurements were recorded, impressions were retaken, and models were cast.

#### 2.1.2. Connective Tissue Graft Harvesting

**Figure 1:**
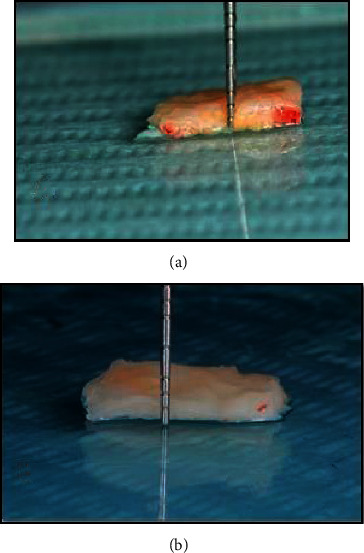
Clinical photo shows (a) harvested epithelialized free gingival graft. (b) Deepithelialized free gingival graft.

The palatal sites were anaesthetized with 0.3 ml of a solution of 4% Articaine and 0.001% Adrenalin **Septanest®**. A rolling test was applied, and keratinized tissue width was measured prior to any surgical intervention in both groups. Free (epithelialized) gingival graft was harvested in both groups by basic surgical techniques previously described by Zucchelli et al. [17], where two horizontal incisions were performed (coronal incision was performed 1–1.5 mm apical to the soft tissue margin of the adjacent teeth), and two vertical incisions were traced to delineate the area to be grafted. Along the coronal horizontal incision, the blade was oriented almost perpendicular to the bone plate, and once an adequate soft tissue thickness was obtained, it was rotated to be almost parallel to the superficial surface. The thickness of the graft was maintained uniform while proceeding apically with the blade, and care was taken not to remove the periosteum protecting the underlying bone. Once the graft was separated, the fatty tissue (yellow in color) was eliminated, and the graft was deepithelialized with a 15°C blade. The graft was positioned on a sterile gauze or a surgical cloth, and its surface was made wet with a saline solution. A light was oriented to be perpendicular to the graft. The different consistency (epithelium is harder and rougher, while the connective tissue is softer and smoother) allowed removal of the epithelium when cutting with the blade kept parallel to the external surface, and the different light reflection (the epithelium reflects more than the connective tissue) enabled us to clinically distinguish when the epithelium was removed. See Figure 1. For all cases, the harvested free gingival graft was of 1.5 mm, and the produced deepithelialized connective tissue graft was of 1 mm thickness.

#### 2.1.3. Control Group

**Figure 2:**
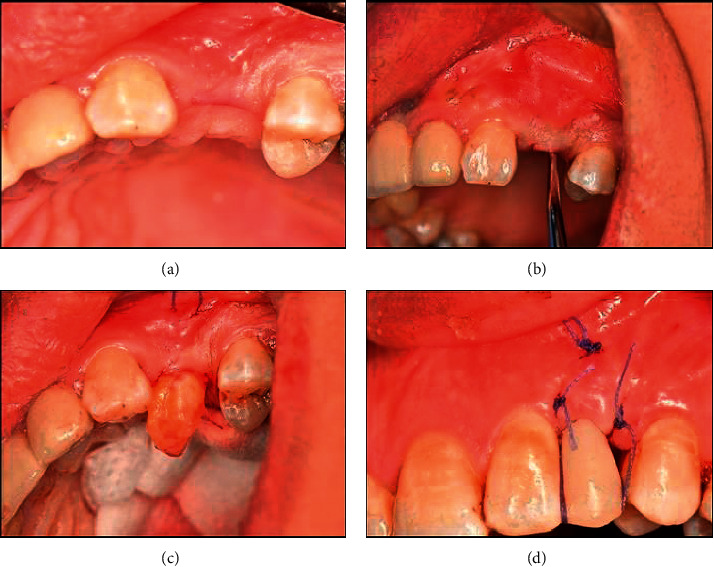
Clinical Photographs showing pouch technique in control group: (a) crestal incision in the site of missing upper left 1^st^ premolar for pouch preparation, (b) prepared pouch after the extension of the dissection beyond the mucogingival line, (c) pull of the de-epithelialized free gingival graft into the pontic site through the crestal incision in the pouch prepared using positional sutures, (d) Provisional restoration is temporarily cemented and double cross suture are applied, and (e) after six months and final bridge is delivered.

Surgical site preparation for pouch technique (Langer and Calagna) [18]. A deep supraperiosteal soft tissue pouch was prepared by sharp dissection extending apically to the mucogingival line and to the neighboring teeth mesiodistally, and the connective tissue graft was inserted into the pouch that was previously prepared at the recipient site through the crestal incision and was secured with two resorbable sutures. Provisional restoration was temporary cemented on both abutments, and then double-crossed sutures were used for the fixation of the buccal soft-tissue complex, including the connective tissue graft. See Figure 2. Patients were recalled after 14 days for suture removal. After six months, the patients delivered fixed prosthesis.

#### 2.1.4. Test Group

**Figure 3:**
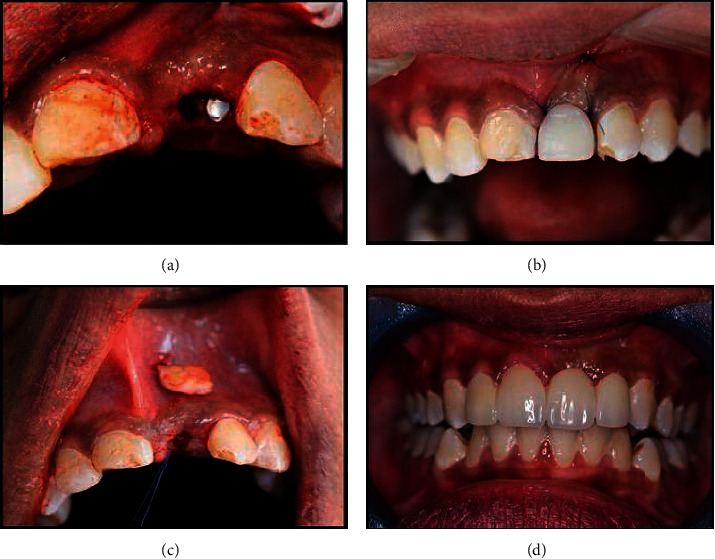
Clinical photographs showing modified pouch technique in test group. (a) Sibert class I ridge defect in the site of missing upper left central incisor, (b) modified pouch with the vertical incision beyond the mucogingival junction, (c) insertion of the connective tissue graft into the pouch from the vertical incision using positioning suture, and (d) final bridge was delivered after 6 months.

Modified pouch technique as described by Zuhr et al. [16]. Incision for tunnel preparation was done in the soft tissue buccal to the pontic site using split thickness flap preparation, as well as the preparation of the pouch extended in a lateral direction to adjacent teeth and in an apical direction beyond the mucogingival line to ensure sufficient flap mobility. Flap preparation included elevation of the interproximal tissues lateral to the edentulous site. Vertical releasing incision in the alveolar mucosa apical and distant to the defect was done. The graft was then drawn into the pouch using positioning sutures through the vertical incision. Provisional restoration was temporarily cemented on both abutments, and then double-crossed sutures were used for the fixation of the buccal soft-tissue complex, including the connective tissue graft. See Figure 3. Patients were recalled after 14 days for suture removal. After six months, the patients delivered fixed prosthesis.

#### 2.1.5. Volumetric Changes

**Figure 4:**
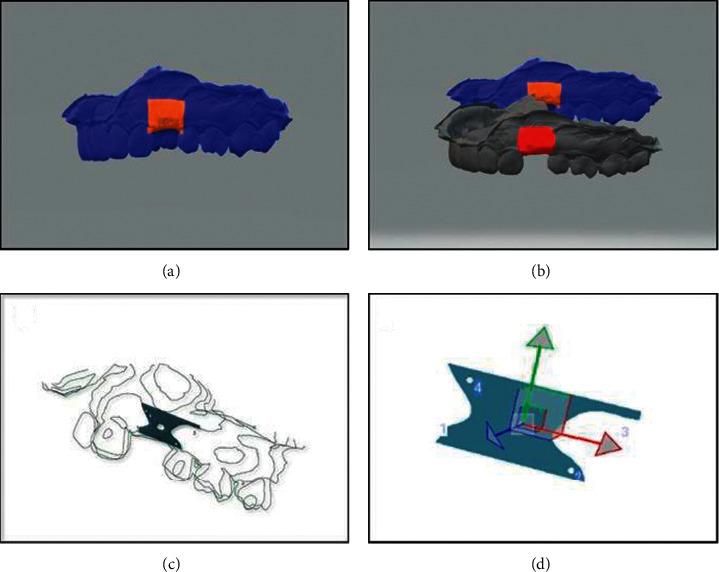
Digital models showing volumetric analysis. (a) Digital model showing determination of the fixed points on the scanned cast to determine the pontic site, (b) digital models showing the alignment of the two pontic sites of the preoperative cast (baseline) and cast poured 3 months post surgically, (c) digital diagram showing volumetric changes calculation in superimposed pontic sites, and (d) digital model showing Isolated section of the pontic site to calculate the area and volume of the site.

After the surgical volume augmentation procedure, impressions were retaken using polyvinyl siloxane impressions (Express 2, 3M Espe), and models were cast at 3 and 6 months postoperatively, in both control and intervention groups, to evaluate the volumetric changes between the baseline and 6 months postoperatively. All cast stone models were digitized using three-dimensional 3D laser scanner (D250, 3Shape). Digital cast models were reproduced, resembling different time points during treatment. The stl files obtained from each model subsequently were transferred to a digital shape sampling and processing software for reelaboration of 3D models from the 3D scan data (Geomagic Studio, Geomagic). For each patient, presurgical and postsurgical models were superimposed, based on a procedure that relies on best matching of manually selected surfaces. The area of pontic site was defined by the mesial and distal papillary midline, the mucogingival line, and the alveolar crest (lateral view and occlusal view of the algorithmic cast superimpositions). The software can then perform an automatic alignment and superimposition in one coordinate system of the two models, based on the best match of these selections. Best-fit alignment used 300 randomly selected points to get an initial orientation, and after superimposition, the marked superimposed pontic sites were isolated, and volumetric changes were between digitalized superimposed casts that were measured and calculated according to Akcali et al. [10]. See Figure 4.

#### 2.1.6. Postoperative Pain

Pain score reported by the patient directly through Visual Analogue Scale (VAS) score (between 0 and 10.0: no pain, 1: minimal pain, 5: moderate pain, 10: severe pain) was recorded at day 3, day 7, and day 14 (Yıldırım et al.) [19].

#### 2.1.7. Keratinized Tissue Thickness

**Figure 5:**
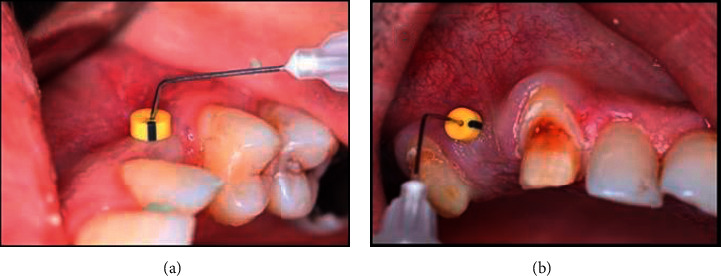
Clinical photos showing measuring keratinized tissue thickness. (a) Needle piercing the keratinized mucosa to measure keratinized tissue thickness at 2 mms and (b) needle piercing the keratinized mucosa to measure keratinized tissue thickness at 4 mms.

This was measured using an anesthetic needle with a rubber stopper, transgingivally piercing tissue horizontally perpendicular to the long axis of the tooth until it contacts the bone at 9 different points: three readings mesially, mid-buccally, and distally at two levels, which were 2 mm, 4 mm, and 6 mm apical to the gingival margin. Th**e** length of the part of the needle that penetrated into the soft tissue was measured using endodontic ruler in mm (Wiesner et al.) [20]. See Figure 5. In this study, there was only one pontic site with more than 6 mm keratinized tissue width, so the readings of this site were of no statistical value.

## 3. Results

### 3.1. Demographic Data

- The study population in this randomized, controlled, double-blinded, parallel-grouped clinical trial included twenty-two patients with single tooth gaps (pontic sites) in the maxillary esthetic area presenting Seibert Class I ridge defects.
- Patients were randomly assigned into two equal groups: test group (n: 11): having a single tooth gap (pontic site) received soft tissue augmentation with connective tissue graft using the modified pouch technique, and control group (n: 11): having a single tooth gap (pontic site) received soft tissue augmentation with connective tissue graft using pouch technique.
- Only sixteen patients completed their follow-up period (8 cases in each group).
- All subjects pontic sites were healed eventually with no complications recorded.
- The data of all subjects examined and completed their follow-ups in the present study were recorded, tabulated, and subjected to statistical analysis and presented in tables and figures. This study was reported according to CONSORT guidelines (Schulz et al) [21].

**Figure 6:**
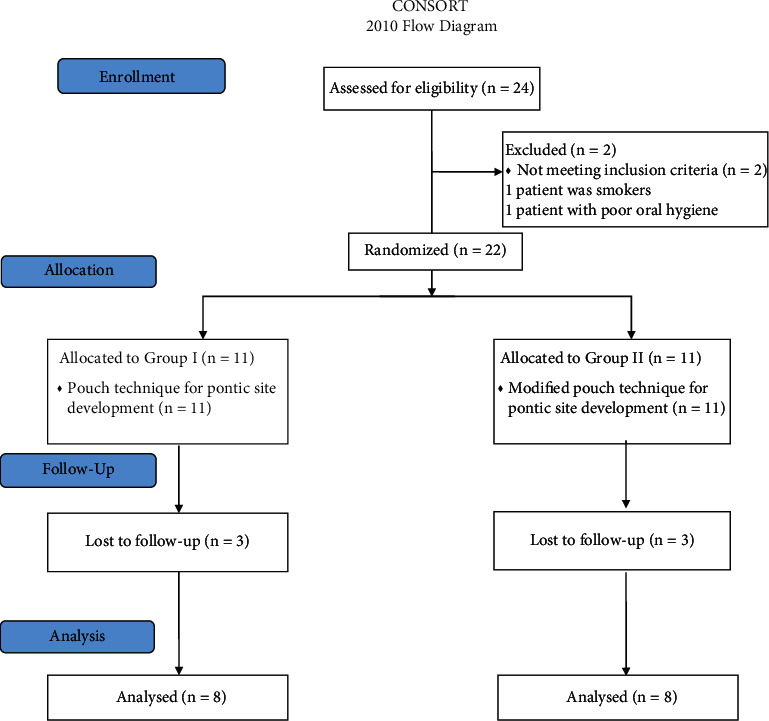
CONSORT flow diagram.

In the test group, there were 2 (25%) males and 6 (75%) females with mean age 36.5 (±4.26). In the control group, there were 4 (50%) males and 4 (50%) females with mean age 34.00 (±5.87) (Figure 6).

### 3.2. Primary Outcome

#### 3.2.1. Volumetric Soft Tissue Changes (mm^3^)

**Figure 7:**
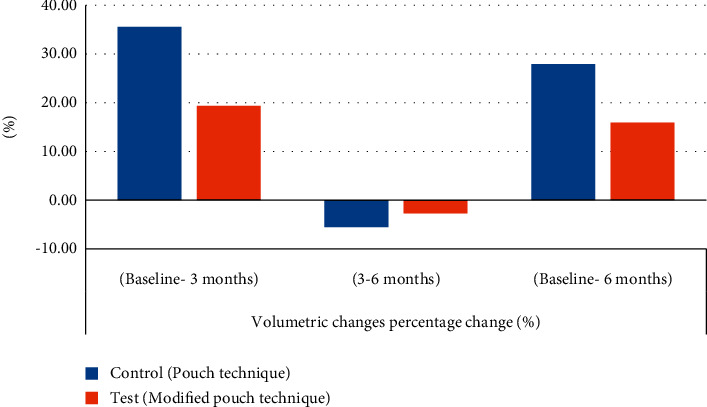
Bar chart showing average values of percent change (%) in soft tissue volumetric changes within each group at different study intervals.

(1) Intragroup comparison: in the test (modified pouch technique), in the control group (pouch technique), there was a statistically significant increase in soft tissue volume (mm^3^) at 3 months as well as at 6 months compared to the baseline values with *p* < 0.001; however, there was a statistically nonsignificant decrease in soft tissue volume (mm^3^) at 6 months compared to 3 months (Table 1)

(2) Intergroup comparison: through all study periods, the test group had a higher soft tissue volume than the control group, yet the difference was not statistically significant (*p*=0.339) (Table 1).

(3) Percent change %: the percent change was calculated between intervals (baseline–3-month interval), (3-month–6-month interval), and (baseline–6-month interval). Regarding the test group, from baseline to 3 months, there was a significant increase in soft tissue volume 19.35 (±16.79) %, while from 3 to 6 months, there was an insignificant reduction in soft tissue volume −2.70 (±1.81) %, and from baseline to 6 months, there was 15.89 (±14.00) % increase in soft tissue volume (Figure 7).

Regarding the control group, from baseline to 3 months, there was a significant increase in soft tissue volume 35.58 (±31.15)%, while from 3 to 6 months, there was an insignificant reduction in soft tissue volume −5.54 (±2.92)%, and from baseline to 6 months, there was 27.94 (±29.20)% increase in soft tissue volume **(**Figure 7**).**

Intergroup comparison:

- From baseline to 3 months, as well as from baseline to 6 months, the control group had a higher percent increase (35.58 (±31.15)) and 27.94 (±29.20) % in soft tissue volume than the test group 19.35 (±16.79) and 15.89 (±14.00) %, yet the difference was not statistically significant with (*p*=0.216) and (*p*=0.310), respectively.
- From 3 to 6 months, the control group had a significantly higher reduction in soft tissue volume −5.54 (±2.92)% than the test group −2.70 (±1.81)% with (*p*=0.034).

### 3.3. Secondary Outcomes

#### 3.3.1. Postoperative Pain (VAS)

*(1)* Intragroup comparisons: regarding the test group, there was a statistically insignificant reduction in pain score (vas) from day 3 to day 7 as well as from day 7 to day 14, while there was a statistically significant reduction in pain score (vas) at day 14 compared to day 3 with *p*=0.001, where no pain was detected at all (table 2).

Regarding the control group, there was a statistically insignificant reduction in pain score (VAS) from day 3 to day 7 as well as from day 7 to day 14, while there was a statistically significant reduction in pain score (VAS) at day 14 compared to day 3 with *p*=0.005, where no pain was detected (Table 2).

Intergroup comparison:

- At day 3, the test group had a higher value (4.62 ± 2.56) than the control group (4.25 ± 3.15), yet the difference was not statistically significant (*p*=0.665)
- At day 7, the control group (3.25 ± 2.71) had a higher value than the test group (2.62 ± 1.51), yet the difference was not statistically significant (*p*=0.450)
- At day 14, both groups had the same mean value (0.00 ± 0.00)

#### 3.3.2. Keratinized Tissue Thickness (KTT) at 2 mm and 4 mm

(1) Intragroup comparisons: Regarding the test group, at 2 mm as well as at 4 mm, there was a significant increase in average KTT at 3 months compared to baseline, while an insignificant reduction was found at 6 months compared to 3 months (Table 3). However, there was a statistically significant increase in KTT at 6 months compared to the baseline value with *p* < 0.001.

The same was observed in the control group, where at 2 mm, as well as at 4 mm, there was a significant increase in average KTT at 3 months compared to baseline, while an insignificant decrease was found at 6 months compared to 3 months. However, there was a statistically significant increase in KTT at 6 months compared to the baseline value with *p* < 0.001.

(2) Intergroup comparison: there was a nonsignificant difference in average KTT at 2 mm, as well as at 4 mm, between the test and control groups throughout the study period (Table 4).

(1) Percentage change %:

Test group:
  - At 2 mm, from baseline to 3 months, there was a significant increase in KTT (77.08 (±36.85)) %, with insignificant reduction from 3 to 6 months that was (−2.78 (±9.21)) %, while from baseline to 6 months, there was a significant increase in KTT (70.42 (±28.14))%.
  - At 4 mm, from baseline to 3 months, there was a significant increase in KTT (138.13 (±52.64)) %, with an insignificant reduction from 3 to 6 months that was −9.93 (±11.07)%, while from baseline to 6 months, there was a significant increase in KTT 115.83 (±61.89)%.
Control group:
  - At 2 mm, from baseline to 3 months, there was a significant increase in KTT (86.88 (±57.75)%), with an insignificant reduction from 3 to 6 months 5.92 (±18.27)%, while there was a significant increase in KTT from baseline to 6 months (70.00 (±43.90)%).
  - At 4 mm, from baseline to 3 months, there was a significant increase in KTT (129.17 (±51.95)%), with an insignificant reduction from 3 to 6 months (5.99 (±9.61) %), while from baseline to 6 months, there was a significant increase in KTT (112.50 (±38.06)%).

## 4. Discussion

The assessment of soft tissue volume is receiving increased attention in the evaluation of treatment outcomes. Although traditional methods such as photographs, direct visual assessment, and transgingival probing have limitations due to reduced accuracy, practicability, and clinical indications, digital optical scanning has been lately applied with the aim of measuring volumetric changes of oral soft tissues over time in a noninvasive way following therapy [22], allowing for a quantitative three-dimensional (3D) assessment of soft tissue changes (González-Martín et al. [23]). Accordingly, the volumetric evaluation performed in this study was carried out by taking impressions at baseline, 3 and 6 months after the surgery, scanning the casts to produce stl files, which were then analyzed using a three-dimensional (3D) laser scanner, which is a noninvasive method, is free of radiation, and does not require much equipment to calculate the pontic site different volumes throughout the treatment procedure.

Results of the present study showed that, in the control group (pouch technique), there was a statistically significant increase in soft tissue volume at 3 months with a 35.58 (±31.15)% change as well as at 6 months with a 27.94 (±29.20)% change, compared to the baseline value with *p* < 0.001; however, there was a statistically nonsignificant decrease in the soft tissue volume at 6 months compared to 3 months with a % change of −5.54 (±2.92)%.

Similarly, in the test group (modified pouch technique), there was a statistically significant increase in the soft tissue volume at 3 months with a 19.35 (±16.79) % change, as well as at 6 months with a 15.89 (±14.00)% change compared to the baseline values with *p* < 0.001; however, there was statistically nonsignificant decrease in soft tissue volume at 6 months compared to 3 months with a % change of −2.70 (±1.81)%.

Intergroup comparison of volumetric soft tissue changes revealed that the test group had an increase in soft tissue volume 175.67 (±57.12) mm^3^ more than control group 145.19 (±65.76) mm^3^ at baseline. The test group also recorded more increase in soft tissue volume 203.44 (±50.65) mm^3^ than control group 182.75 (±58.00) mm^3^ at 3 months. Similar results were also registered at 6 months where the test group had more increase in soft tissue volume 198.17 (±50.39) mm^3^ than control group 173.51 (±57.84) mm^3^.

The control group had a higher percent change from baseline to 3 months as well as from baseline to 6 months (35.58 (±31.15) and 27.94 (±29.20)%) than the test group (19.35 (±16.79) and 15.89 (±14.00) %), yet the difference was not statistically significant with *p*=0.216 and *p*=0.310, respectively, while from 3 to 6 months, the control group had a significantly more % change −5.54 (±2.92)% than the test group −2.70 (±1.81)% with *p*=0.034, which indicates that the loss of the initially gained volume was significantly more in the control group than the test group.

This loss of the initially gained volume at 6 months could be attributed to the shrinkage of the connective tissue graft used in soft tissue augmentation [6]. It is noteworthy that the graft shrinkage in the present study occurred only between 3 and 6 months. This finding is somehow surprising, because the most pronounced alterations are usually anticipated to occur within the first 3 months. However, most available clinical trials were conducted on shorter terms and did not include observation periods of the same duration as in the present clinical trial. Thus, we support the recommendation of postponing final restorative measures until after 6 months of augmentation procedures, due to the qualitative and possibly quantitative alterations that might occur during this time due to maturation [24].

To the best of the authors' knowledge, this is the first clinical trial that examined volume alterations after soft tissue augmentation using pouch versus modified pouch technique; thus, it is very difficult to correlate our results. However, in some respects, the results of the present study could be correlated with the few studies that tackled the field of soft tissue augmentation in pontic site development or those studies in which volumetric changes after soft tissue grafting were assessed.

The present study is also in line with a previous prospective study conducted by Gonzalez et al. that performed pontic site development in the esthetic zone using subepithelial connective tissue graft by pouch technique to assess volumetric gain. A quantitative three-dimensional (3D) analysis based on laser scanning was used for the measurement of volume gain and horizontal changes of the alveolar profile 5 months after surgery in five patients. All surgical sites healed uneventfully. A mean soft tissue volume increase of 35.9 mm^3^ was measured 5 months after the grafting procedure. The linear measurements showed that, in the area where the augmentation was performed, the distance between the preoperative vestibular profile and the postoperative one ranged from 0.16 to 2 mm in the grafted areas [23].

Furthermore, the result of the present study correlates with a randomized controlled clinical trial introduced by Akcali et al. to compare the volumetric changes in pontic site development with either vascularized interpositional periosteal connective tissue grafts (VIPCG) or subepithelial connective tissue grafts in Seibert class 1 ridge defects [10]. The outcomes demonstrated a contour change in labial distance between baseline and the 6-month follow-up of 1.2 mm for VIPCG sites and 0.6 mm for SCTG sites. In addition, a loss of the initially gained volume was reported, amounting to 47% at 6 months for SCTG sites and to 6.4% for VIPCG sites, showing significantly less shrinkage of the graft.

Additionally, the present study correlates with case series done by Narayan et al. [25] who used interpositional graft in conjunction with a provisional ovate pontic in the maxillary esthetic zone to achieve an ideal esthetic restoration. Three months postoperatively, there was an increase in the horizontal dimension in the deficient ridge and an esthetic emergence profile.

In the present study, keratinized tissue thickness was measured using an anesthetic needle with a rubber stopper, transgingivally piercing tissue horizontally perpendicular to the long axis of the tooth until it contacts the bone at 9 different points: three readings mesially, mid-buccally, and distally at three different levels 2 mm, 4 mm, and 6 mm apical to the gingival margin. In the present study, only 2 cases had keratinized tissue width >6 mm; thus, readings of keratinized tissue thickness at 6 mm were excluded from the statistical analysis. The length of the part of the instrument that penetrates the soft tissue was measured in mm [20].

Keratinized tissue thickness at 2 mm was increased from baseline to 3 months in both test and control groups in the mesial, middle, and distal sides and from 3 to 6 months only on the mesial side. On the other hand, a reduction in keratinized tissue thickness at 2 mm was observed from 3 to 6 months in the middle and distal side in the control group and only in the middle aspect in the test group with a −6.25 (±28.08)% change. The intergroup comparison of keratinized tissue thickness at 2 mm showed that the mesially test group showed higher mean values than the control group at baseline, 3 and 6 months, yet the difference was not statistically significant. Both groups had the same mean percent change value from baseline to 3 months and from baseline to 6 months, while the control group had a higher percent change value from 3 to 6 months. In contrast, on the middle aspect, the control group demonstrated higher mean values than test group, yet the difference was not statistically significant at baseline and 3 months, while both groups had the same mean value at 6 months. The control group had a higher percent change value than the test group from baseline to 3 months, whereas the control group recorded more percent reduction (−17.71 (±20.14)%) in keratinized tissue thickness than the test group (−4.17 (±11.79)%) from 3 to 6 months. Thus, more loss of keratinized tissue thickness at 2 mm was observed on the middle aspect, and this was more pronounced in the control group.

For the distal aspect, both groups had the same mean value at baseline and 3 months, while the test group had a higher mean value than the control group at 6 months. Both groups had the same percent change value from baseline to 3 months, while the test group recorded no percent change from 3 to 6 months, while in the control group, there was a percent reduction −4.17 (±11.79)% in the initially gained keratinized tissue thickness. Regarding the average readings, the test group had a higher mean value than the control group at baseline and 6 months, while both groups had the same mean value at 3 months. The control group demonstrated more percent reduction in keratinized tissue thickness at 2 mm −5.92 (±18.27)% than the test group −2.78 (±9.21)% from 3 to 6 months.

Regarding keratinized tissue thickness at 4 mm, the intergroup comparison showed that mesially both groups showed the same mean values at baseline and 3 months, while the control group had a higher mean value than the test group at 6 months. The control group showed more percent change from baseline to 3 months and from baseline to 6 months, while the test group demonstrated more percent reduction in keratinized tissue thickness at 4 mm than the control group from 3 to 6 months.

On the middle aspect, both groups had the same mean value at baseline, while the control group demonstrated a higher mean values than the test group at 3 and 6 months. The control group had a higher percent increase value than the test group from baseline to 3 months and from baseline to 6 months; meanwhile, the control group recorded more percent reduction than the test group from 3 to 6 months.

For the distal aspect, the control group recorded higher mean values than the test group at baseline, 3 and 6 months, yet the difference was not statistically significant. The test group recorded more percent increase than the control group from baseline to 3 months, while the control group showed less percent reduction keratinized tissue thickness at 4 mm than the test group from 3 to 6 months. Both groups had the same percent change value from baseline to 6 months.

Regarding average readings, the control group also demonstrated higher mean values than the test group at baseline, 3 and 6 months. The test group demonstrated more percent increase of keratinized tissue thickness at 4 mm than the control group from baseline to 3 months and from baseline to 6 months. On the contrary, the test group demonstrated more percent reduction −9.93 (±11.07) and demonstrated more percent reduction −5.99 (±9.61) % from 3 to 6 months.

Moreover, a split-mouth randomized controlled clinical trial was done to evaluate whether connective tissue grafts performed at implant placement could be effective in augmenting peri-implant soft tissues. The trial was performed on ten partially edentulous patients requiring at least one single implant in the premolar or molar areas of both sides of the mandible and randomized to have one side augmented at implant placement with a connective soft tissue graft harvested from the palate or no augmentation; they stated that soft tissues at augmented sites were 1.3 mm thicker (*P* < 0.001) [20].

Our results also correlate with a recent systematic review conducted by Zucchelli et al. [26], which stated that soft tissue augmentation of deformed ridges in the esthetic zone can dramatically improve the aesthetics of the restoration. They used three different soft tissue augmentation techniques, connective tissue graft (CTG), keratinized gingival graft (KGG), and pediculated connective tissue graft (PCTG) to treat severe ridge deformities in the maxillary anterior region in five patients. They demonstrated that all three soft tissue grafting techniques demonstrated long-term stability following the six-month posttreatment result.

Moreover, the present results are in line with Thoma et al. [27] who performed a randomized controlled clinical trial to test whether or not the use of a collagen matrix (VCMX) would result in short-term soft tissue volume increase at implant sites comparable to an autogenous subepithelial connective tissue graft (SCTG) and to evaluate safety and tissue integration of VCMX and SCTG. In 20 patients with volume deficiency at single-tooth implant sites, soft tissue volume augmentation was performed randomly allocating VCMX or SCTG. Median soft tissue thickness increased by 1.5 mm (1.0; 2.0) (SCTG) (*p*=0.395) after 90 days' of the surgery as recorded.

In the present study, both groups were tested for the pain scores reported by the patient directly through Visual Analogue Scale (VAS) score (between 0 and 10.0: no pain, 1: minimal pain, 5: moderate pain, 10: severe pain) was recorded at day 3, day 7, and day 14 [19].

In the test group, there was a statistically insignificant reduction in pain score (VAS) from day 3 to day 7 as well as from day 7 to day 14, while there was a statistically significant reduction in pain score (VAS) at day 14 compared to day 3 with *p*=0.001 where no pain was detected. Likewise, the control group showed a statistically insignificant reduction in pain score (VAS) from day 3 to day 7 as well as from day 7 to day 14, while there was a statistically significant reduction in pain score (VAS) at day 14 compared to day 3 with *p*=0.005, where no pain was detected.

The intergroup comparison demonstrated a higher pain score mean value (4.62 ± 2.56) in the test group than the control group (4.25 ± 3.15) at day 3, yet the difference was not statistically significant (*p*=0.665). On the other hand, the control group (3.25 ± 2.71) had a higher mean value than the test group (2.62 ± 1.51) at day 7, yet the difference was not statistically significant (*p*=0.450), while at day 14, both groups had the same mean value (0.00 ± 0.00), denoting that there is no significant different in postoperative pain between the two techniques.

These results were in accordance with a randomized controlled clinical trial by Thoma et al. [27] whi reported a slightly higher VAS score for SCTG between day 1 and day 3 after surgery without showing any statistically significant difference at any time point (*p* > 0.05), while it was diminished on day 14 with suture removal.

## 5. Conclusions

Soft tissue augmentation using both the traditional pouch technique and the modified pouch technique led to successful soft tissue volume augmentation and a successful increase in keratinized tissue thickness in pontic site development in Seibert Class I ridge defects. Graft shrinkage in the present study occurred between 3 and 6 months. Thus, we support the recommendation of postponing final restorative measures until after 6 months of augmentation procedures.

## Figures and Tables

**Table 1:** Mean and SD values for comparison in volumetric soft tissue changes (mm^3^) between both groups at different study periods.

Interval	Volumetric changes (mm^3^) mean (±SD)		*p* value
	Test (Modified pouch technique)	Control (Pouch technique)	
Baseline	175.67 (±57.12)^B^	145.19 (±65.76)^B^	0.339 ns
3 months	203.44 (±50.65)^A^	182.75 (±58.00)^A^	0.460 ns
6 months	198.17 (±50.39)^A^	173.51 (±57.84)^A^	0.379 ns
*p*-value	<0.001^*∗*^	<0.001^*∗*^	

Means with different superscript letters within the same horizontal row are significantly different. ^*∗*^; significant (*p* ≤ 0.05) ns; nonsignificant (*p* > 0.05)

**Table 2:** Mean and SD values of post-operative pain (VAS) within each group at days 3, 7, and 14

Group	Postoperative pain (VAS) mean (±SD)			*p* value
	Day 3	Day 7	Day 14	
Test (Modified pouch technique)	4.62 (±2.56)^A^	2.62 (±1.51)^AB^	0.00 (±0.00)^B^	0.001^*∗*^
Control (Pouch technique)	4.25 (±3.15)^A^	3.25 (±2.71)^AB^	0.00 (±0.00)^B^	0.005^*∗*^
*p*-value	0.665ns	0.450ns	NA	

Means with different superscript letters within the same horizontal row are significantly different. NA: Not Applicable, ^*∗*^; significant (*p* ≤ 0.05) ns; nonsignificant (*p* > 0.05).

**Table 3:** Mean and SD values of keratinized tissue thickness at 2 mm and 4 mm within each group at different study periods.

Point	Study Period	Groups		*p* value
		Test	Control	
Keratinized tissue thickness at 2 mm	Baseline	1.58 (±0.35)^B^	1.54 (±0.40)^B^	0.826 ns
	3 months	2.71(±0.33)^A^	2.71(±0.42)^A^	1.000 ns
	6 months	2.63 (±0.33)^A^	2.50 (±0.36)^A^	0.479 ns
	*p*-value	<0.001^*∗*^	<0.001^*∗*^	

Keratinized tissue thickness at 4 mm	Baseline	1.29 (±0.38)^B^	1.37 (±0.28)^B^	0.622 ns
	3 months	2.92 (±0.24)^A^	3.04 (±0.38)^A^	0.438 ns
	6 months	2.62 (±0.38)^A^	2.83 (±0.18)^A^	0.178 ns
	*p*-value	<0.001^*∗*^	<0.001^*∗*^	

Means with different superscript letters within the same horizontal row are significantly different. ^*∗*^; significant (*p* ≤ 0.05) ns; nonsignificant (*p* > 0.05)

**Table 4:** Mean and SD values of keratinized tissue thickness (KTT) at 2 mm and 4 mm percentage change (%) within each group at different study periods.

Point	Study Period	Groups		*p* value
		Test	Control
Keratinized tissue thickness at 2 mm	Baseline–3 months	77.08 (±36.85)^A^	86.88 (±57.75)^A^	0.692 ns
	3 months–6 months	−2.78 (±9.21)^B^	−5.92 (±18.27)^B^	0.670 ns
	Baseline–6 months	70.42 (±28.14)^A^	70.00 (±43.90)^A^	0.982 ns
	P value	<0.001^*∗*^	<0.001^*∗*^	

Keratinized tissue thickness at 4 mm	Baseline–3 months	138.13 (±52.64)^A^	129.17 (±51.95)^A^	0.737 ns
	3 months–6 months	−9.93 (±11.07)^B^	−5.99 (±9.61)^B^	0.460 ns
	Baseline–6 months	115.83 (±61.89)^A^	112.50 (±38.06)^A^	0.899 ns
	P value	<0.001^*∗*^	<0.001^*∗*^	

Means with different superscript letters within the same horizontal row are significantly different ^*∗*^; significant (*p*  ≤ 0.05) ns; nonsignificant (*p* > 0.05)

## Data Availability

The data that support the findings of this study are available at the Outpatient Clinics of the Faculty of Dentistry, Cairo University, and restrictions apply to the availability of these data, which were used under license for the current study, and so are not publicly available. Data, however, are available from the corresponding author upon reasonable request.
